# Supplementary material for: HIVprotI: an integrated web based platform for prediction and design of HIV proteins inhibitors
Source: J Cheminform. 2018 Mar 9;10:12. doi: 10.1186/s13321-018-0266-y (PMC5845081; doi:10.1186/s13321-018-0266-y)
Supplement: Supplementary file 2 — Additional file 2. Source code of HIVProtI web server. [file 13321_2018_266_MOESM2_ESM.zip › HIVprotI_Source-code/hivprotI/weka-3-6-6/doc/weka/estimators/Estimator.html]

Estimator


---


|  |  |  |
| --- | --- | --- |
| **Overview** \| **Package** \| **Class** \| **Tree** \| **Deprecated** \| **Index** \| **Help** \| **Weka's home** | |  |
| **PREV CLASS**   **NEXT CLASS** | **FRAMES**    **NO FRAMES**     **All Classes** |  |
| SUMMARY: NESTED \| FIELD \| CONSTR \| METHOD | DETAIL: FIELD \| CONSTR \| METHOD |  |


---


## weka.estimators Class Estimator

```
java.lang.Object
  weka.estimators.Estimator
```

**All Implemented Interfaces:**: java.io.Serializable, java.lang.Cloneable, CapabilitiesHandler, OptionHandler, RevisionHandler

**Direct Known Subclasses:**: DiscreteEstimator, DiscreteEstimatorBayes, KernelEstimator, MahalanobisEstimator, NormalEstimator, PoissonEstimator

---

``` public abstract class Estimator extends java.lang.Object implements java.lang.Cloneable, java.io.Serializable, OptionHandler, CapabilitiesHandler, RevisionHandler ```

Abstract class for all estimators.
Example code for a nonincremental estimator
 ```` ```
   // create a histogram for estimation
   EqualWidthEstimator est = new EqualWidthEstimator();
   est.addValues(instances, attrIndex);
``` ```` 
Example code for an incremental estimator (incremental
estimators must implement interface IncrementalEstimator)
 ```` ```
   // Create a discrete estimator that takes values 0 to 9
   DiscreteEstimator newEst = new DiscreteEstimator(10, true);

   // Create 50 random integers first predicting the probability of the
   // value, then adding the value to the estimator
   Random r = new Random(seed);
   for(int i = 0; i < 50; i++) {
     current = Math.abs(r.nextInt() % 10);
     System.out.println(newEst);
     System.out.println("Prediction for " + current 
                        + " = " + newEst.getProbability(current));
     newEst.addValue(current, 1);
   }
``` ```` 
Example code for a main method for an estimator.

```` ```
 public static void main(String [] argv) {

   try {
     LoglikeliEstimator est = new LoglikeliEstimator();      
     Estimator.buildEstimator((Estimator) est, argv, false);      
     System.out.println(est.toString());
   } catch (Exception ex) {
     ex.printStackTrace();
     System.out.println(ex.getMessage());
   }
 }
``` ```` 

**Version:**
:   $Revision: 5539 $

**Author:**
:   Gabi Schmidberger (gabi@cs.waikato.ac.nz), Len Trigg (trigg@cs.waikato.ac.nz)

**See Also:**: Serialized Form

---

**Constructor Summary**

`Estimator()`


| **Method Summary** | |
| --- | --- |
| `void` | `addValue(double data, double weight)`             Add a new data value to the current estimator. |
| `void` | `addValues(Instances data, int attrIndex)`             Initialize the estimator with a new dataset. |
| `void` | `addValues(Instances data, int attrIndex, double min, double max, double factor)`             Initialize the estimator with all values of one attribute of a dataset. |
| `void` | `addValues(Instances data, int attrIndex, int classIndex, int classValue)`             Initialize the estimator using only the instance of one class. |
| `void` | `addValues(Instances data, int attrIndex, int classIndex, int classValue, double min, double max)`             Initialize the estimator using only the instance of one class. |
| `static void` | `buildEstimator(Estimator est, Instances instances, int attrIndex, int classIndex, int classValueIndex, boolean isIncremental)` |
| `static void` | `buildEstimator(Estimator est, java.lang.String[] options, boolean isIncremental)`             Build an estimator using the options. |
| `static Estimator` | `clone(Estimator model)`             Creates a deep copy of the given estimator using serialization. |
| `java.lang.String` | `debugTipText()`             Returns the tip text for this property |
| `boolean` | `equals(java.lang.Object obj)`             Tests whether the current estimation object is equal to another estimation object |
| `static Estimator` | `forName(java.lang.String name, java.lang.String[] options)`             Creates a new instance of a estimatorr given it's class name and (optional) arguments to pass to it's setOptions method. |
| `Capabilities` | `getCapabilities()`             Returns the Capabilities of this Estimator. |
| `boolean` | `getDebug()`             Get whether debugging is turned on. |
| `java.lang.String[]` | `getOptions()`             Gets the current settings of the Estimator. |
| `abstract  double` | `getProbability(double data)`             Get a probability estimate for a value. |
| `java.util.Enumeration` | `listOptions()`             Returns an enumeration describing the available options. |
| `static Estimator[]` | `makeCopies(Estimator model, int num)`             Creates a given number of deep copies of the given estimator using serialization. |
| `static Estimator` | `makeCopy(Estimator model)`             Creates a deep copy of the given estimator using serialization. |
| `void` | `setDebug(boolean debug)`             Set debugging mode. |
| `void` | `setOptions(java.lang.String[] options)`             Parses a given list of options. |
| `void` | `testCapabilities(Instances data, int attrIndex)`             Test if the estimator can handle the data. |

**Methods inherited from class java.lang.Object**

`getClass, hashCode, notify, notifyAll, toString, wait, wait, wait`

**Methods inherited from interface weka.core.RevisionHandler**

`getRevision`

**Constructor Detail**

### Estimator

```
public Estimator()
```


**Method Detail**

### addValue

```
public void addValue(double data,
                     double weight)
```

:   Add a new data value to the current estimator.

    :   **Parameters:**: `data` - the new data value: `weight` - the weight assigned to the data value

---


### addValues

```
public void addValues(Instances data,
                      int attrIndex)
               throws java.lang.Exception
```

:   Initialize the estimator with a new dataset.
    Finds min and max first.

    :   **Parameters:**: `data` - the dataset used to build this estimator: `attrIndex` - attribute the estimator is for **Throws:**: `java.lang.Exception` - if building of estimator goes wrong

---


### addValues

```
public void addValues(Instances data,
                      int attrIndex,
                      double min,
                      double max,
                      double factor)
               throws java.lang.Exception
```

:   Initialize the estimator with all values of one attribute of a dataset.
    Some estimator might ignore the min and max values.

    :   **Parameters:**: `data` - the dataset used to build this estimator: `attrIndex` - attribute the estimator is for: `min` - minimal border of range: `max` - maximal border of range: `factor` - number of instances has been reduced to that factor **Throws:**: `java.lang.Exception` - if building of estimator goes wrong

---


### addValues

```
public void addValues(Instances data,
                      int attrIndex,
                      int classIndex,
                      int classValue)
               throws java.lang.Exception
```

:   Initialize the estimator using only the instance of one class.
    It is using the values of one attribute only.

    :   **Parameters:**: `data` - the dataset used to build this estimator: `attrIndex` - attribute the estimator is for: `classIndex` - index of the class attribute: `classValue` - the class value **Throws:**: `java.lang.Exception` - if building of estimator goes wrong

---


### addValues

```
public void addValues(Instances data,
                      int attrIndex,
                      int classIndex,
                      int classValue,
                      double min,
                      double max)
               throws java.lang.Exception
```

:   Initialize the estimator using only the instance of one class.
    It is using the values of one attribute only.

    :   **Parameters:**: `data` - the dataset used to build this estimator: `attrIndex` - attribute the estimator is for: `classIndex` - index of the class attribute: `classValue` - the class value: `min` - minimal value of this attribute: `max` - maximal value of this attribute **Throws:**: `java.lang.Exception` - if building of estimator goes wrong

---


### getProbability

```
public abstract double getProbability(double data)
```

:   Get a probability estimate for a value.

    :   **Parameters:**: `data` - the value to estimate the probability of **Returns:**: the estimated probability of the supplied value

---


### buildEstimator

```
public static void buildEstimator(Estimator est,
                                  java.lang.String[] options,
                                  boolean isIncremental)
                           throws java.lang.Exception
```

:   Build an estimator using the options. The data is given in the options.

    :   **Parameters:**: `est` - the estimator used: `options` - the list of options: `isIncremental` - true if estimator is incremental **Throws:**: `java.lang.Exception` - if something goes wrong or the user requests help on command options

---


### buildEstimator

```
public static void buildEstimator(Estimator est,
                                  Instances instances,
                                  int attrIndex,
                                  int classIndex,
                                  int classValueIndex,
                                  boolean isIncremental)
                           throws java.lang.Exception
```

:   **Throws:**: `java.lang.Exception`

---


### clone

```
public static Estimator clone(Estimator model)
                       throws java.lang.Exception
```

:   Creates a deep copy of the given estimator using serialization.

    :   **Parameters:**: `model` - the estimator to copy **Returns:**: a deep copy of the estimator **Throws:**: `java.lang.Exception` - if an error occurs

---


### makeCopy

```
public static Estimator makeCopy(Estimator model)
                          throws java.lang.Exception
```

:   Creates a deep copy of the given estimator using serialization.

    :   **Parameters:**: `model` - the estimator to copy **Returns:**: a deep copy of the estimator **Throws:**: `java.lang.Exception` - if an error occurs

---


### makeCopies

```
public static Estimator[] makeCopies(Estimator model,
                                     int num)
                              throws java.lang.Exception
```

:   Creates a given number of deep copies of the given estimator using serialization.

    :   **Parameters:**: `model` - the estimator to copy: `num` - the number of estimator copies to create. **Returns:**: an array of estimators. **Throws:**: `java.lang.Exception` - if an error occurs

---


### equals

```
public boolean equals(java.lang.Object obj)
```

:   Tests whether the current estimation object is equal to another
    estimation object

    :   **Overrides:**: `equals` in class `java.lang.Object`
    :   **Parameters:**: `obj` - the object to compare against **Returns:**: true if the two objects are equal

---


### listOptions

```
public java.util.Enumeration listOptions()
```

:   Returns an enumeration describing the available options.

    :   **Specified by:**: `listOptions` in interface `OptionHandler`
    :   **Returns:**: an enumeration of all the available options.

---


### setOptions

```
public void setOptions(java.lang.String[] options)
                throws java.lang.Exception
```

:   Parses a given list of options. Valid options are:

    -D   
    If set, estimator is run in debug mode and
    may output additional info to the console.

    :   **Specified by:**: `setOptions` in interface `OptionHandler`
    :   **Parameters:**: `options` - the list of options as an array of strings **Throws:**: `java.lang.Exception` - if an option is not supported

---


### getOptions

```
public java.lang.String[] getOptions()
```

:   Gets the current settings of the Estimator.

    :   **Specified by:**: `getOptions` in interface `OptionHandler`
    :   **Returns:**: an array of strings suitable for passing to setOptions

---


### forName

```
public static Estimator forName(java.lang.String name,
                                java.lang.String[] options)
                         throws java.lang.Exception
```

:   Creates a new instance of a estimatorr given it's class name and
    (optional) arguments to pass to it's setOptions method. If the
    classifier implements OptionHandler and the options parameter is
    non-null, the classifier will have it's options set.

    :   **Parameters:**: `name` - the fully qualified class name of the estimatorr: `options` - an array of options suitable for passing to setOptions. May be null. **Returns:**: the newly created classifier, ready for use. **Throws:**: `java.lang.Exception` - if the classifier name is invalid, or the options supplied are not acceptable to the classifier

---


### setDebug

```
public void setDebug(boolean debug)
```

:   Set debugging mode.

    :   **Parameters:**: `debug` - true if debug output should be printed

---


### getDebug

```
public boolean getDebug()
```

:   Get whether debugging is turned on.

    :   **Returns:**: true if debugging output is on

---


### debugTipText

```
public java.lang.String debugTipText()
```

:   Returns the tip text for this property

    :   **Returns:**: tip text for this property suitable for displaying in the explorer/experimenter gui

---


### getCapabilities

```
public Capabilities getCapabilities()
```

:   Returns the Capabilities of this Estimator. Derived estimators have to
    override this method to enable capabilities.

    :   **Specified by:**: `getCapabilities` in interface `CapabilitiesHandler`
    :   **Returns:**: the capabilities of this object **See Also:**: `Capabilities`

---


### testCapabilities

```
public void testCapabilities(Instances data,
                             int attrIndex)
                      throws java.lang.Exception
```

:   Test if the estimator can handle the data.

    :   **Parameters:**: `data` - the dataset the estimator takes an attribute from: `attrIndex` - the index of the attribute **Throws:**: `java.lang.Exception` **See Also:**: `Capabilities`


---


|  |  |  |
| --- | --- | --- |
| **Overview** \| **Package** \| **Class** \| **Tree** \| **Deprecated** \| **Index** \| **Help** \| **Weka's home** | |  |
| **PREV CLASS**   **NEXT CLASS** | **FRAMES**    **NO FRAMES**     **All Classes** |  |
| SUMMARY: NESTED \| FIELD \| CONSTR \| METHOD | DETAIL: FIELD \| CONSTR \| METHOD |  |


---
